# Elimination of Delamination during the Drilling of Biocomposite Materials with Flax Fibers

**DOI:** 10.3390/polym16182620

**Published:** 2024-09-16

**Authors:** Martin Váňa, Štěpánka Dvořáčková, Tomáš Knápek, Dora Kroisová

**Affiliations:** Assembly and Engineering Metrology, Department of Machining, Faculty of Mechanical Engineering, Technical University of Liberec, 461 17 Liberec, Czech Republic

**Keywords:** biocomposite materials, drilling, delamination, cutting tool wear, backing plates

## Abstract

The present study focuses on the elimination of delamination during the drilling of a linen-based biocomposite material in epoxy resin used for the manufacture of sports kayaks, depending on the tool material, cutting conditions, and the use of additional wooden support plates. In the present study, HSS (high-speed steel) and Carbide cutting tools without coatings, with the same geometry and two types of cutting conditions (n = 1500 rpm, f_n_ = 0.05 and 0.1 mm/rev) were used. A Sololite-type wooden backing plate was used to aid in reducing delamination. The results show that the additional support plates significantly reduced delamination by up to 80% both at the material inlet and especially at the drill hole outlet. In this study, the use of a lower feed rate (f_n_ = 0.05 mm/rev) per tooth was shown to have a significant effect on reducing the delamination of biocomposite materials with flax fibers, which are generally known to be difficult to machine. The Carbide cutting tool shows significantly better results both in terms of its wear and in terms of delamination of the biocomposite material. The highest delamination was obtained without the use of a backing board at the tool exit after 50 drilled holes of 3509 µm. With the use of a backing board, this delamination decreased to 693 µm after 50 drilled holes.

## 1. Introduction

Biocomposite materials (hereinafter BCMs) are currently finding significant applications in a wide range of industrial and service sectors, particularly in terms of reducing the environmental impact of these products. The most commonly used reinforcements in these systems are flax fibers or flax fabrics, which have mechanical parameters close to those of synthetically produced fibers, in particular glass fibers. The mechanical parameters of flax fibers are very promising compared with synthetic fibers and offer many possibilities, especially in the current focus on minimizing the environmental impact of materials and technological processes [1,2,3,4,5].

Epoxy resins are still preferably used as matrices for composite systems. The principal disadvantage of reinforcing flax fibers is mainly their wettability, which is due to their chemical composition. For this reason, such materials must be processed and treated to meet the requirements for a specific application. In the manufacture of parts from BCMs, it is very difficult to make holes, cut-outs, grooves, etc., without affecting the fiber system; as the resin matrix is crushed, the fibers are exposed, and the ends fray at the point of contact with the tool [6,7]. 

When machining composite materials, the reinforcement profile affects the performance of the machining process. Here, the profile represents the arrangement of the fibers, their volume fraction, and the fiber architecture. Because of the anisotropic response of the system, composite materials show a significant difference in machining performance compared with homogeneous materials/metals because at least two diametrically different materials (matrix and reinforcement) are machined simultaneously. For these reasons, investigating the machining response of BCMs is very complicated. Several problems are encountered, usually caused by the high strength of the reinforcement, leading to rapid wear of the cutting tool assembly. BCMs/laminates are made of many layers, which practically predisposes them to the delamination process that results from the destruction of the polymer matrix and the simultaneous pulling out of the reinforcing fibers. The high absorbency of the reinforcing fibers largely limits the use of conventional process fluids in the machining process [8,9,10,11].

Current trends in the use of environmentally acceptable materials such as BCMs are leading to the need to address technological processes such as machining. It is estimated that up to 60% of all scrapped parts are due to poor hole quality, and as the holes are drilled in the last stage of production, scrapping parts because of poor hole quality leads to large economic losses. These defects arising in the machining process can be evaluated by direct observation and control of the resulting damage (delamination, surface roughness, micro-cracks, matrix or fiber burn-out, etc.) depending on the machining conditions [12]. The quality of the machined surface or delamination of BCMs depends on process parameters such as the cutting speed, feed rate, material, geometry of the cutting tool, etc. The choice of optimum process parameters is very important to obtain a high-quality workpiece. For this reason, it is essential to study their responses (delamination, surface roughness, cutting tool wear, etc.) [13,14].

Various studies, such as Belaadi et al. [10] investigated the delamination factor in drilling BCMs (jute/polyester; 5.7 and 10 mm wood drill) and the subsequent optimization of the process to reduce the resulting delamination. The delamination factor was found to be most influenced by the feed rate, reinforcement fiber length, and drill bit diameter. The delamination factor increased with a higher fiber length, higher feed rate, and larger drill bit diameter. Another factor found to be influential is the method of manufacturing the composite laminate. Higher porosity leads to higher delamination. Tool wear has a large influence on the resulting hole quality and is related to the appropriate geometry and material of the cutting tool. Different cutting materials or coatings can lead to different results for a given workpiece. The work by Benyettou [9] investigated the behavior of BCMs (date palm fiber/polyester) drilled with helical HSS drills with coatings (HSS-TiN, HSS-C, and HSS-Co). The best hole quality with respect to delamination was achieved with the HSS-Co drill bit. On the other hand, optimum roundness was achieved with the HSS-TiN drill bit. The best cylindricity was then obtained with the HSS-C drill. Furthermore, the work by Tabeta et al. [15] investigated the effect and optimization of process parameters on BCMs (bi-directional jute fiber and cork-reinforced polymer) during drilling (helical HSS-TiN drill and HSS wood drill; both drill bits had d = 5.7 and 10 mm). They found that the importance of material and the feed rate in relation to drill diameter had a predominant effect on the delamination factor. It was also found that spindle speed had no effect on this factor. The contribution of each element to the optimum drilling conditions was as follows: feed rate (66.04%), drill diameter (10.54%), and then spindle speed.

As aforementioned, a major issue in machining BCMs is delamination. Delamination can be reduced by using backing plates. Backing plates can be used in industrial practice to reduce damage caused by drilling and to ensure good quality of the resulting hole at both the inlet and outlet of the hole [10,16]. The material of the backing plates can be selected according to the characteristics of the material to be machined. The correct choice will reduce delamination, but the wrong choice of backing plate material (thickness, surface roughness, and material) may have the opposite effect and lead to increased delamination or other damage to the work material or increased wear on the cutting tool. Inappropriately selected insert parameters will contribute to higher cutting tool wear, leading to lower efficiency and increased costs [17,18]. 

This study investigates the effect of backing plates on the amount of delamination produced at the inlet and outlet of the drilled hole. This study is divided into inter-related parts evaluating the effect of the number of holes on cutting tool wear, the effect of the worn tool on the size of delamination, and the elimination of delamination by backing plates. The importance and subsequent applicability of this study are related to the behavior of natural cellulose-based fiber materials, whose major disadvantage is their wettability. Wettability will be a major problem in delamination; the frayed fibers of the flax reinforcement will absorb water and change the mechanical and physical parameters of the component. Verifying the suitability of the choice of reinforcement in accordance with its wear resistance, determining the process conditions, and simultaneously minimizing delamination by using backing plates has significant potential in the machining capabilities of these environmentally friendly materials.

The results of this initial study open further questions about the possibility of machining these materials by other methods [19,20].

## 2. Materials and Methods

**Figure 1:**
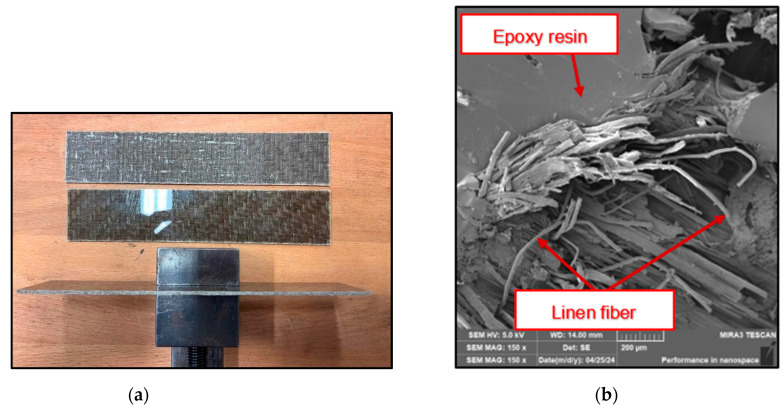
Machined BCM: (**a**) sample of the composite material plate and (**b**) part of the delaminated surface after drilling with loose flax fibers.

The biocomposite material (BCM) used for this experiment was laminate boards from a Czech manufacturer—Figure 1. The reinforcing fabric is made of long flax fibers with a 2/2 twill weave. The technical parameters are given in Table 1. The matrix material is an epoxy resin with the designation LG 700 (GRM Systems, Olomouc, Czech Republic). The advantages of this resin include extremely low viscosity, which allows the formation of very low-weight laminates, high reactivity, processing time (25 min to 180 min), good temperature resistance even after curing at room temperature, and high flexibility, which is maintained while maintaining excellent temperature resistance. Technical parameters and mechanical properties are given in Table 2 and Table 3. It is a material used in the manufacture of some primary and secondary parts of aircraft and sports kayaks. The laminates were cut into test specimens of 300 mm × 150 mm × 2 mm (length × width × specimen thickness).

### 2.1. Cutting Tool and Cutting Parameters

**Figure 2:**
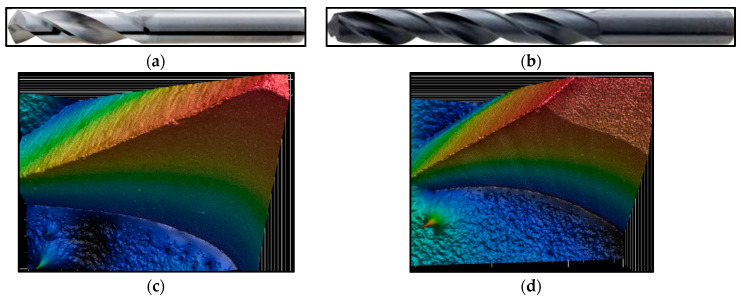
Drilling tools: (**a**) HSS drill, (**b**) Carbide drill, (**c**) 3D scan of the HSS cutting edge, and (**d**) 3D scan of the Carbide cutting edge.

The cutting tools were 2 helical drills with a diameter of 6 mm with a tip angle of 118°; these were HSS and Carbide drills without surface treatment, as shown in Table 4 and Figure 2. 

Furthermore, two spindle speed levels (500 and 1500 rpm) and two feed rates per revolution (0.05 and 0.1 mm/rev) were determined for this study. 

To simulate a real industrial process, machining without process media was performed, as shown in Table 5.

These tools were chosen because of their price, availability on the market, and also because they are the most commonly used tools for conventional machining of composite systems, which were also to be tested for biocomposite materials to see if their performance and properties would suit this specific type of material.

### 2.2. Machining Machine

The drilling process was carried out on a 3-axis CNC milling machine from the manufacturer DMG MORI type CMX 600V (DMG Mori Seiki, Nagoya, Japan) with a maximum spindle speed capacity of 12,000 rpm.

**Figure 3:**
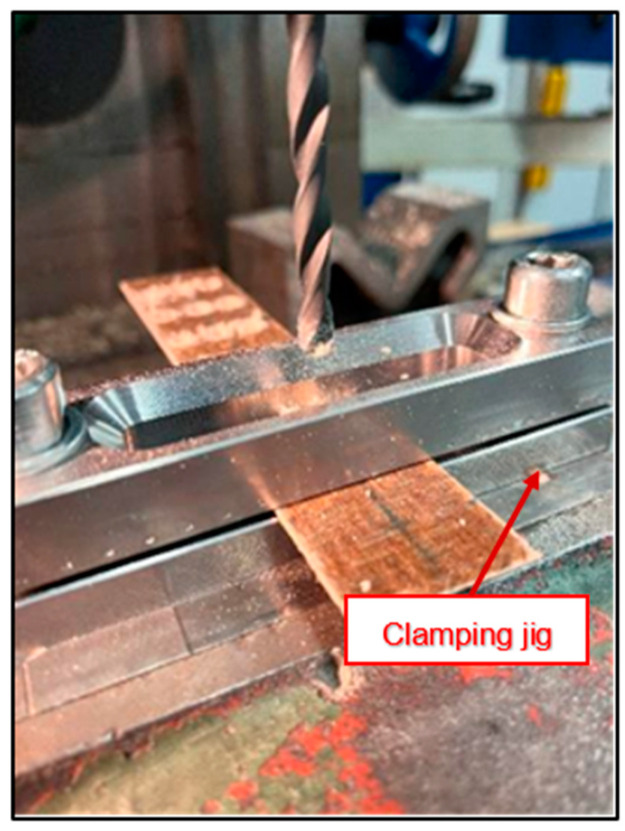
Clamping jig.

The samples were clamped in a special jig, as shown in Figure 3, because of the small thickness of the BCM (2 mm), which cannot be clamped in a conventional machine vice.

### 2.3. Support Plate

**Figure 4:**
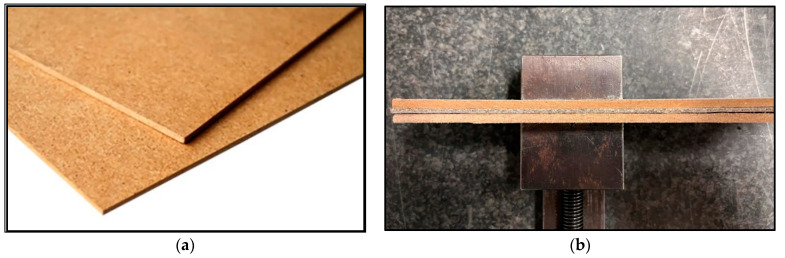
Support plates: (**a**) Sololite support plate and (**b**) fixing the sample between the plates.

The support board chosen for this study was a Sololite-type fiberboard. The dimensions of the board were 300 mm × 150 mm × 3.2 mm (length × width × specimen thickness), as shown in Figure 4. The specimen was placed between two support plates to keep it stable during drilling. The thickness of the plates was deliberately chosen to be higher to ensure the system’s stability.

### 2.4. Methods for Evaluating Tool Wear and the Delamination of Samples 

The analysis/measurement of the amount of cutting tool wear and delamination was carried out using a Keyence VK-X 1000 (Keyence, Itasca, IL, USA) confocal microscope. The measurements were always performed after a fixed number of drilled holes. The measurement interval was determined after drilling the 1st, 10th, 30th, 40th, and 50th holes. The Keyence VK-X 1000 confocal microscope was used to assess cutting tool wear by measuring the linear change/decay in tool edge dimensions. The confocal microscope was also used to assess the extent of delamination at the inlet and outlet by measuring the change like the sample around the drilled hole.

## 3. Results

Delamination is the most visible and most serious defect that occurs when machining BCMs. Delamination is generally characterized as the separation of layers/laminations of the composite material. In a drilling operation, delamination is distinguished into delamination that occurs at the beginning and end of the hole, i.e., the drill entry and exit. Delamination at the beginning of the hole is the result of torque and may not always be present. Delamination at the end of the hole is the result of the forces generated as the drill bit passes through the material and, unlike entry delamination, is much more noticeable and undesirable as it reduces the quality of the hole, increases the wetting of the system, and has an overall impact on the quality and strength of the composite.

**Figure 5:**
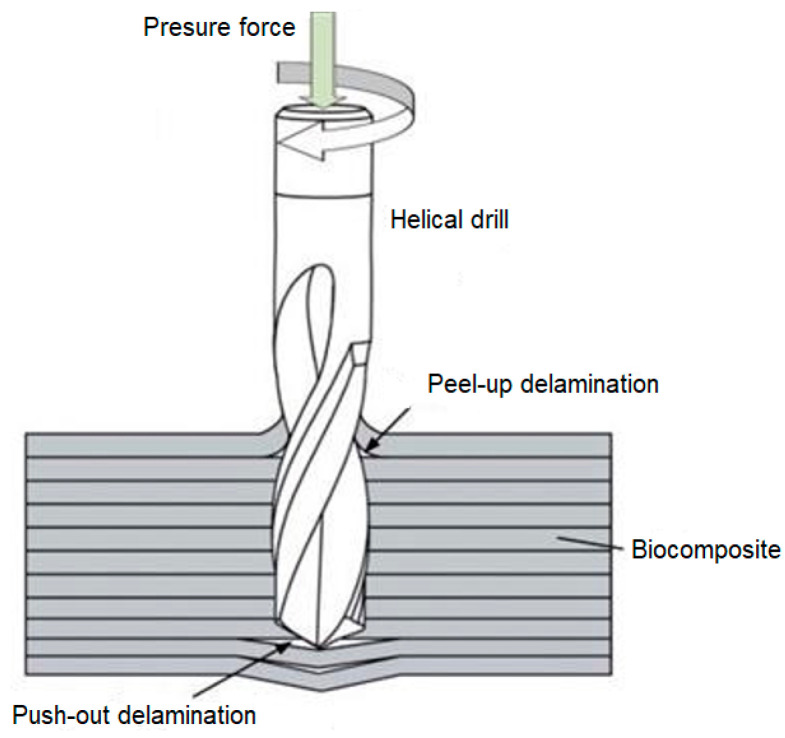
Mechanism of delamination during drilling.

As the drill bit begins to penetrate the composite laminate, the top layer of the laminate breaks and is displaced upwards by the action of the helical drill bit. The fibers are pulled towards the surface of the sample and break the compact top layer of resin. As the cutting tool advances with the downward feed, the worn tool layer helically coils downward with the drill groove. This leads to the drill bit gradually passing through the sample and separating one layer of laminate from the other because of the destruction of both the resin and the fibers. When the transverse blade of the cutting tool reaches the lower layers of the composite laminate, both compressive and tensile forces are applied to the fibers simultaneously with the rotation of the drill bit. The complicated force action on the underside of the specimen and the drill bit entering the free space below the specimen will cause the most noticeable delamination of the composite system Figure 5.

According to the above, delamination is a significant problem when drilling BCMs, so it is important to address the issue and try to eliminate this defect, which subsequently causes a poor quality/poor drilled hole. This study focused on the elimination of delamination of drilled holes using backing plates when using HSS and Carbide cutting tools without surface treatment at the given cutting conditions shown in Table 5. The amount of wear on the cutting tool was measured on both the main and secondary cutting edges. In terms of the BCM delamination analysis, the amount of delamination at both the hole inlet and the hole outlet was measured for each hole. To evaluate the measured results, it was necessary to establish a limiting value for the average magnitude of FD_crit_ delamination at the inlet and outlet of the drilled hole. There is no industry standard or prescriptive standard for this limit value. In this study, a limiting average value of FD_crit_ = 1500 µm was chosen.

### 3.1. Effect of the Number of Holes on Cutting Tool Wear

The measured results shown in Table 6 indicate that at cutting conditions n = 1500 rpm and f_n_ = 0.10 mm/rev, there was higher wear than at n = 1500 rpm and f_n_ = 0.05 mm/rev for both cutting tools for both the main and secondary cutting edges. For HSS, the wear was more pronounced on the secondary blade, while for Carbide, the wear was more pronounced on the main blade. The wear increased continuously with an increasing number of holes. 

After drilling 50 holes with the HSS tool, the wear reached the highest values of 16.30 µm and 18.39 µm for n = 1500 rpm and f_n_ = 0.10 mm/rev for the main and secondary cutting edges, as shown in Table 7. The HSS tool gradually failed to cut the threads at the exit of the hole, which led not only to higher delamination and damage to the holes but also to further wear of the secondary blade.

After drilling 50 holes with the Carbide tool, the highest wear was also achieved at cutting conditions of n = 1500 rpm and f_n_ = 0.10 mm/rev, with values of 8.64 µm and 5.77 µm for the main and secondary blades, respectively. The first wear was also observed after drilling 30 holes on the main blade and 40 holes on the secondary blade. However, the cutting tool did not show abrasion wear like the HSS tool.

### 3.2. Effect of the Number of Holes on Delamination

Based on the measured results, the amount of tool wear affected the amount of delamination. The magnitude of delamination increased with cutting tool wear and with higher feed per revolution, i.e., with cutting conditions n = 1500 rpm and f_n_ = 0.10 mm/rev. The delamination was most pronounced at the outlet holes, as shown in Table 8.

Compared with other composite materials, BCMs are characterized by lower hardness and lower abrasiveness because of the reinforcing flax fibers used. These properties, together with the small thickness of the test specimens used in this study, result in minimal tool wear and therefore minimal effect on the magnitude of delamination at both the inlet and outlet of the hole. 

The displacement per revolution has a significant effect on the overall size of the delamination of the drilled hole. The explanation may be that as the feed rate increases, the magnitude of the compressive force of the cutting tool also increases. The cutting tool pushes on the work material, causing matrix cracking, the bending of loose fibers, and the formation of delamination, including its growth. 

After drilling 50 holes with the HSS tool, the highest value of delamination was obtained for cutting conditions n = 1500 rpm and f_n_ = 0.10 mm/rev, and the values at the exit of the hole were 3509 µm and, at the entrance, 1990 µm.

After drilling 50 holes with the Carbide tool, the highest delamination value was also obtained for cutting conditions n = 1500 rpm and f_n_ = 0.10 mm/rev, and the values at the exit hole were 2077 µm and, the entrance, 942 µm. 

The delamination at the hole exit was always higher than the hole entry for both tools, as shown in Table 9. The delamination increased with higher feed per revolution, i.e., with cutting conditions n = 1500 rpm and f_n_ = 0.10 mm/rev. The low feed rate f_n_ = 0.05 mm/rev contributed to reducing the magnitude of delamination.

### 3.3. Effect of Support Plates on the Size of Delamination

The use of wooden backing plates resulted in a significant reduction in the amount of delamination at both the inlet and outlet of the drilled hole for both cutting tools, as shown in Table 9. 

The wooden support plates, because of their properties as a firm but relatively soft material, were able to adhere firmly to the drilled material, thus preventing fiber deflection before cutting, but also reducing the delamination values of the laminate layers, as shown in Table 10.

For the cutting conditions n = 1500 rpm and f_n_ = 0.05 mm/rev, the wooden backing plates, because of their properties, allowed for reducing the size of delamination for the HSS cutting tool from 3012 µm to 611 µm at the exit and from 1181 µm to 359 µm at the entrance of the drilled hole. For the Carbide cutting tool, the amount of delamination decreased from 1442 µm to 228 µm at the exit and from 597 µm to 125 µm at the entrance of the hole. 

At the cutting conditions n = 1500 rpm and f_n_ = 0.1 mm/rev, the delamination of the HSS cutting tool was also reduced when using a wooden backing plate, from 3509 µm to 693 µm at the hole exit and from 1990 µm to 579 µm at the hole entry. For the Carbide cutting tool, the size of delamination also decreased rapidly at the exit from 2077 µm to 299 µm and at the hole entrance from 942 µm to 176 µm. 

Because of the use of wooden backing plates, both cutting tools were able to meet the given limiting value of average delamination size (FD_krit_ = 1500 µm).

## 4. Discussion

BCMs are now becoming popular because of their favorable cost, weight, and environmental character. They are used for the manufacture of special automotive and aerospace components, bicycle frames, window frames, sports equipment, etc. 

BCMs exhibit a weakness at the level of interfacial adhesion between the fibers and matrix, which is related to the high hydrophilicity of natural fibers due to their chemical composition and the hydrophobic nature of synthetic polymer matrices. A further complication is their unstable chemical, physical, and mechanical properties compared with synthetic fibers, which is due to the variable proportion of cellulose, hemicellulose, lignin, and other substances affected by plant growth conditions. Other limitations include the range of processing temperatures, low impact resistance, and low thermal stability. The variability in their properties is due to their high dependence on the time and conditions of harvest, the climate of the agricultural site, the soil and weather, agricultural practices, cultivation techniques and plant conditions, mechanical extraction, and bundle production. These disadvantages negatively affect the long-term durability of BCMs, limiting their use to non-structural and semi-structural components (e.g., door panels, dashboards, sports equipment) and thus slowing their integration into high-performance applications. Any modification (drilling, milling, etc.) of BCM components requires a specific approach to designing the machining conditions so that the mechanical properties of BCMs are not significantly compromised.

BCMs are characterized by low abrasion and overall lower hardness compared with other composite materials (e.g., carbon fiber reinforced plastic—CFRP). 

For this reason, low wear rates were observed for both the HSS and Carbide cutting tools in the present study. The wear for both cutting tools increased with the number of holes drilled. Further, the wear was dependent on the given cutting conditions. The magnitude of wear reached higher values with higher feed per revolution, i.e., with f_n_ = 0.10 mm/rev. For HSS, the wear reached 16.30 µm for the main cutting edge and 18.39 µm for the secondary cutting edge after 50 holes were drilled. For Carbide, it was 8.64 µm for the main blade and 5.77 µm for the secondary blade.

According to the analysis of the results, the lower feed per revolution f_n_ = 0.05 mm/rev showed to be the best in terms of tool life for both types of tools, i.e., HSS and Carbide. For HSS, it was 13.59 µm for the main blade and 14.39 µm for the secondary blade, and for Carbide, it was 5.66 µm for the main blade and 4.55 µm for the secondary blade, as shown in Table 6 and Table 7.

The improved performance of the Carbide tool is due to the material composition of the drill bit surface, which resists the most corrosive types of materials. Working with natural fibers is very complicated because of the material composition of the fibers and, at the same time, the structure of the fiber, which is itself a composite.

The amount of delamination was measured at the entrance and exit of the drilled hole depending on the cutting conditions. In the experimental measurements, it was shown that as the feed rate increases from f_n_ = 0.05 to 0.1 mm/revolution, the amount of delamination increases both at the inlet and especially at the outlet of the drilled hole. The magnitude of delamination at the outlet was always recorded to be larger than that at the inlet of the drilled hole. 

After drilling 50 holes, the highest value of delamination was obtained for cutting conditions n = 1500 rpm and f_n_ = 0.10 mm/rev for both the HSS- and Carbide-type tools. After drilling 50 holes with the HSS tool, the highest delamination value obtained at the exit hole was 3509 µm and at the entrance was 1990 µm, and for Carbide, the high value at the exit hole was 2077 µm and at the entrance was 942 µm. The delamination at the exit of the holes was always higher than at the entrance of the holes for both tools, as shown in Table 9. The lower feed per revolution value f_n_ = 0.05 mm/rev again came out as the best for both types of cutting tools according to the delamination analysis. For HSS, the delamination value at the hole exit after drilling 50 holes was 3012 µm and at the hole entry was 1181 µm, and for Carbide, at the hole exit, it was 1442 µm and at the hole entry, it was 597 µm.

Only the Carbide tool met the predefined threshold value of average delamination size, FD_crit_ = 1500 µm. The HSS tool failed to meet the limit value. The results obtained are in agreement with the data in the publications by Belaadi et al. [5,6] and Tabeta et al. [4].

The displacement per revolution has a significant effect on the overall size of the delamination of the drilled hole. As the feed rate increases, i.e., f_n_ = 0.05 to f_n_ = 0.10 mm/rev, the size of the delamination increases. The explanation may be that as the feed rate increases, the magnitude of the compressive force of the cutting tool also increases. As the cutting tool pushes on the workpiece, the workpiece defends itself against cutting, matrix cracking, fiber bending, and delamination including its growth. 

The micro-cracks caused by drilling are generated when subjected to tension, fatigue loading, or different temperature conditions. Interlaminar cracks, fiber–matrix cracks, and transverse cracks are a group of micro-cracks at the interface. The resulting micro-cracks in the composite progress in the transverse direction and thus, the thickness of the composite significantly affects the mechanical properties of the composite. The formed micro-cracks act as initiators for other types of defects such as delamination, spalling, chipping, or fiber fraying. Fiber collapse occurs because of the presence of moisture in the reinforced fibers of the composite system. Fiber bunching adversely affects the adhesive bond between the fiber and the matrix material. The defects described above lead to interlaminar debonding during the implementation of machining forces. An equally serious problem associated with the drilling of natural fiber composites is the formation of burrs on the surface of the parts caused by delamination of the reinforcing fibers, which subsequently complicates further assembly operations.

Based on this set of facts, it is necessary to minimize the presence of water in biocomposite systems with natural fibers and thus eliminate machining with process fluids. At the same time, delamination at the drill inlet and outlet of the composite system must be minimized.

The experimental measurements showed that the use of backing plates had a significant effect on reducing the magnitude of delamination, especially for both cutting conditions of n = 1500 rpm and f_n_ = 0.05 and 0.1 mm/rev, as shown in Table 9 and Table 10.

For cutting conditions n = 1500 rpm and f_n_ = 0.05 mm/rev, the wooden support plates, because of their properties, allowed for reducing the size of delamination for the HSS cutting tool by about 80% on average at the exit and about 70% at the entrance of the drilled hole. For the Carbide cutting tool, the amount of delamination was reduced by an average of about 84% at the exit and about 79% at the entrance of the drilled hole.

For cutting conditions n = 1500 rpm and f_n_ = 0.1 mm/rev, for the HSS cutting tool, delamination was also reduced with the use of a wooden backing plate by an average of about 80% at the exit and about 71% at the entrance of the drilled hole. For the Carbide cutting tool, there was also a significant reduction in the amount of delamination by about 85% on average at the exit and about 81% at the entrance of the drilled hole.

The use of inexpensive wooden backing plates in the BCM drilling process, compared with the values without the use of backing plates, resulted in a rapid reduction in the magnitude of delamination on the surface of the samples for both cutting tools and cutting conditions. The reduction in delamination at the inlet and outlet of the hole can be explained by the stiffening of the tool–workpiece system, including the jig used. Increasing the stiffness of the machined system is the most important factor according to the experimentally obtained results. Because of the additional plate on the top and bottom of the specimen, pulling the fibers up with the drill bit and pushing the bottom lamin of the composite down is minimized. This reduces pull-up and push-down delamination. Further experiments could be performed regarding the backing plate material and its pressure on the specimen. This issue is underdeveloped, as biocomposites are not yet widely used in engineering practice because of the already-mentioned information on the nature of natural fibers [4].

As a result, both cutting tools met the given limit value for the average size of delamination (FD_crit_ = 1500 µm).

However, it should be pointed out that each BCM is heterogeneous with different manufacturing defects (poor penetration, formation of porosity, etc.) and different material compositions, which are usually influenced by the properties of the fibers used (dependence on harvest, climate, soil, and weather and on agricultural practices, cultivation techniques, and plant conditions) or mechanical treatment of the fibers. The combination of cutting conditions and cutting tools will affect any BCMs during processing, including varying amounts of delamination.

A comprehensive study of delamination is important not only from the point of view of the actual delamination process, which is influenced by the tool and technological parameters, but especially the impact of delamination. Delamination generally results in a reduction in the mechanical parameters of the component, which is generally undesirable. Delamination in the case of biocomposite materials where cellulose-based fibers are used, i.e., fibers that are easily wetted, is even more complicated as the adsorption and absorption of moisture in these materials can also increase the dimensions of the component. Because of the permeation of moisture through the material along/inside the natural fibers at the molecular level and the potential changes that moisture can induce, the stage of this issue is crucial [19,20].

## 5. Conclusions

To study the elimination of BCM delamination (with flax fibers) during the drilling process, two cutting tools with the same geometry but different material compositions (HSS and Carbide) and two types of cutting conditions (n = 1500 rpm, f_n_ = 0.05 and 0.1 mm/rev.) were used. To help reduce delamination, a Sololite-type pressed wood fiber backing plate was used.

The following conclusions were drawn from this study:

- The abrasion wear of the cutting tool increased with an increasing number of drilled holes for both the HSS and Carbide types. Because of the low hardness and abrasiveness of the BCM with flax fibers, the wear was generally low (up to 20 µm for HSS and 10 µm for Carbide) for both types of cutting conditions with a difference in the feed rate per revolution.
- The shape and size of delamination increased with higher feed per revolution, i.e., from f_n_ = 0.05 to 0.1 mm/rev at speed n = 1500 rpm, and with wear of both the HSS- and Carbide-type cutting tools. Higher delamination values were recorded at the drilled hole output because of the low stiffness of the BCM system and the compressive force exerted by the tool with wear on the main and secondary cutting edges (matrix cracking due to fiber bending instead of cutting, fiber stretching by the tool, etc.). The lower feed rate (f_n_ = 0.05 mm/rev) per tooth contributed to the reduction in the delamination of BCMs.
- The wooden support plates rapidly reduced delamination both at the entrance and especially at the exit of the drilled hole. Using cutting conditions n = 1500 rpm and f_n_ = 0.05 mm/rev, this was on average about 80% at the exit and about 70% at the entrance of the drilled hole for the HSS cutting tool, and about 84% at the exit and about 79% at the entrance of the drilled hole for the Carbide cutting tool. For cutting conditions n = 1500 rpm and f_n_ = 0.1 mm/rev, the HSS cutting tool reduced delamination by approx. 80% at the exit and approx. 71% at the drill hole entrance. The Carbide cutting tool reduced delamination by approx. 85% at the exit and approx. 81% at the drill hole entrance.

Based on the results of this study, it is evident that when drilling BCMs with laminate thicknesses in the millimeter range, the use of a wooden backing plate leads to stabilization of the machining process and a reduction in delamination at both the input and output by up to 80%. The use of a lower feed rate (f_n_ = 0.05 mm/rev) per tooth also leads to a reduction in delamination, which is essential when using these materials. The Carbide-type cutting tool material shows much better results in terms of both wear and delamination.

## Figures and Tables

**Table 1:** Technical parameters of linen reinforcement.

Parameter	Value
Area weight of fabric [g/m^2^]	200
Fiber density [kg/dm^3^]	1.47
Tensile modulus of elasticity [GPa]	62
Extension at break [%]	1.3–1.4
Water content [% for 22 °C]	5–6
Weave	Twill 2/2

**Table 2:** Technical parameters of epoxy resin.

Parameter	Value
Density [g/cm^3^]	1.18–1.23
Viscosity [mPa.s]	500–700
Epoxy equivalent [mol/kg]	156–165
Epoxy index [-]	0.6–0.64

**Table 3:** Mechanical properties of cured epoxy resin without reinforcement.

Parameter	Value
Strength limit [MPa]	110–120
E modul [MPa]	2700–3300
Tensile strength limit [MPa]	65–75
Compressive strength limit [MPa]	120–140
Ductility [%]	6–8
Impact toughness [kJ/m^2^]	38–48

**Table 4:** Parameters of cutting tools.

Tool Material	HSS	Carbide
Coating	-	-
Tool diameter [mm]	6	6
Tool length [mm]	57	28
Tip angle [°]	118	118
Helical angle [°]	30	30
Number of cutting edges	2	2

**Table 5:** Cutting conditions.

Cutting Conditions	Value
Spindle speed [rpm]	1500
Feed per revolution [mm/rev]	0.05, 0.1

**Table 6:** Analysis of HSS and Carbide cutting tool wear as a function of the number of drilled holes.

		HSS		Carbide	
Cutting Conditions	Number of Holes	Wear on the Main Blade [µm]	Wear and Tear on Adjacent Blade [µm]	Wear on the Main Blade [µm]	Wear and Tear on Adjacent Blade [µm]
n = 1500 rpm, f_n_ = 0.05 mm/rev	1	0.00	0.00	0.00	0.00
	10	0.00	0.00	0.00	0.00
	30	4.28	7.32	4.49	0.00
	40	8.30	9.00	4.70	3.94
	50	13.59	14.39	5.66	4.55
n = 1500 rpm, f_n_ = 0.1 mm/rev	1	0.00	0.00	0.00	0.00
	10	0.00	0.00	0.00	0.00
	30	7.25	12.48	5.87	0.00
	40	11.55	15.96	7.10	5.09
	50	16.30	18.39	8.64	5.77

The measurement uncertainty was between 0.7 and 0.9 for all values.

**Table 7:** Example of wear on the main cutting edge of the HSS and Carbide cutting tools depending on the number of holes drilled.

Cutting Tool—HSS			
10 holes	(n = 1500 rpm, f_n_ = 0.10 mm/rev)		50 holes
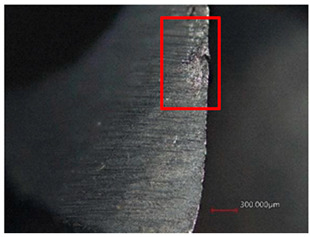		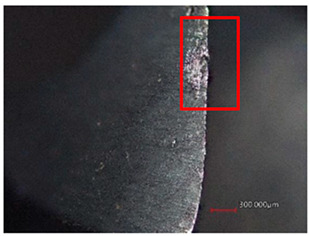	
A defect in a cutting tool is a good indication of its wear mechanism, namely, abrasion. In the red boxes, it is easy to see how the cutting tool back is “polished”—the cutting tool surface becomes increasingly shiny.			
Cutting Tool—Carbide			
10 holes	(n = 1500 rpm, f_n_ = 0.10 mm/rev)		50 holes
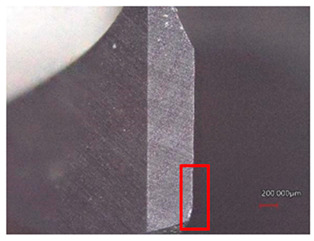		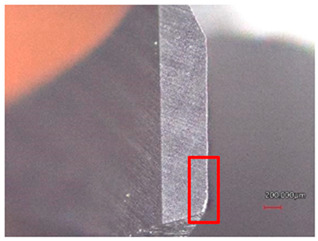	
As with the HSS cutting tool, abrasion wear occurred, as can be seen in the pictures (red boxes).			

**Table 8:** Delamination size analysis for the HSS and Carbide cutting tools.

Cutting Tool—HSS	In	Out
n = 1500 rpm, f_n_ = 0.05 mm/rev	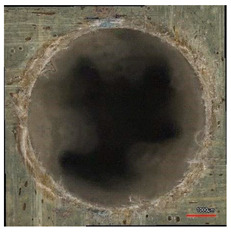	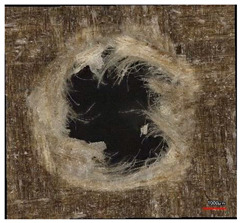
n = 1500 rpm, f_n_ = 0.10 mm/rev	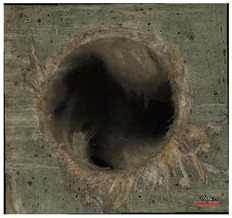	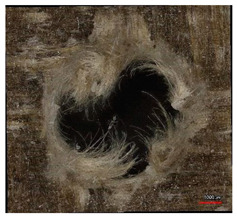
Cutting Tool—Carbide	In	Out
n = 1500 rpm, f_n_ = 0.05 mm/rev	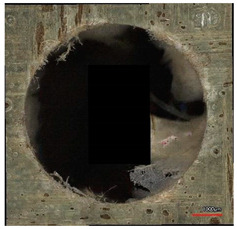	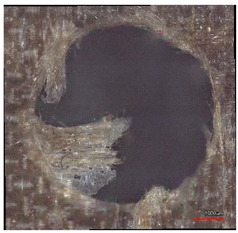
n = 1500 rpm, f_n_ = 0.10 mm/rev	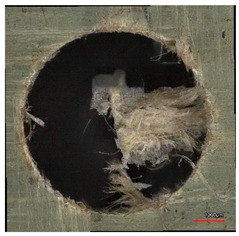	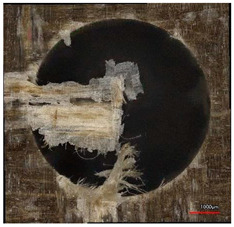

**Table 9:** Analysis of the amount of delamination depending on the used fiberboards for the HSS and Carbide cutting tools.

Cutting Tool		HSS		Carbide	
Cutting Conditions	Number of Holes	Output Delamination [µm]	Input Delamination [µm]	Output Delamination [µm]	Input Delamination [µm]
n = 1500 rpm, f_n_ = 0.05 mm/rev	1	2827	1049	1357	534
	10	2889	1063	1368	537
	30	2959	1118	1343	550
	40	3008	1170	1386	552
	50	3012	1181	1442	597
Wood-fiber backing board	1	560	308	205	106
	10	581	318	211	108
	30	598	329	214	112
	40	602	333	220	115
	50	611	359	228	125
n = 1500 rpm, f_n_ = 0.1 mm/rev	1	3324	1822	1977	913
	10	3379	1886	2002	938
	30	3397	1890	2048	1009
	40	3462	1895	2056	1079
	50	3509	1990	2077	942
Wood fiber backing board	1	655	545	271	164
	10	673	556	274	173
	30	681	565	284	167
	40	688	571	283	172
	50	693	579	299	176

**Table 10:** Example of the size of delamination without and with a wooden backing plate for the Carbide cutting tool.

Cutting Tool—Carbide; n = 1500 rpm, f_n_ = 0.05 mm/rev	
Delamination at the Entrance of the Drilled Hole	
Without support plate	With a wooden support plate
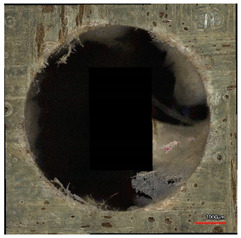	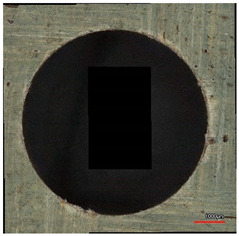
Delamination at the exit of the drilled hole	
Without support plate	With a wooden support plate
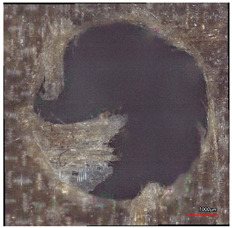	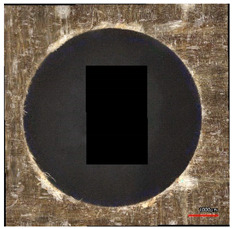

## Data Availability

Data are contained within the article.

## References

[1] Goutianos S., Peijs T., Nystrom B., Skrifvars M. (2006). Development of Flax Fibre Based Textile Reinforcements for Composite Applications. Appl. Compos. Mater..

[2] Zouhar J., Slaný M., Sedlák J., Joska Z., Pokorný Z., Barényi I., Majerík J., Fiala Z. (2022). Application of Carbon–Flax Hybrid Composite in High Performance Electric Personal Watercraft. Polymers.

[3] Oun A., Manalo A., Alajarmeh O., Abousnina R., Gerdes A. (2022). Influence of Elevated Temperature on the Mechanical Properties of Hybrid Flax-Fiber-Epoxy Composites Incorporating Graphene. Polymers.

[4] Ngo T.-D. (2017). Natural Fibers for Sustainable Bio-Composites. Natural and Artificial Fiber-Reinforced Composites as Renewable Sources.

[5] Blanchard J.M.F.A., Sobey A.J. (2019). Comparative Design of E-Glass and Flax Structures Based on Reliability. Compos. Struct..

[6] Musa C., Zaidi M., Depriester M., Allouche Y., Naouar N., Bourmaud A., Baillis D., Delattre F. (2024). Development of Foam Composites from Flax Gum-Filled Epoxy Resin. J. Compos. Sci..

[7] Schuster J., Govignon Q., Bickerton S. (2014). Processability of Biobased Thermoset Resins and Flax Fibres Reinforcements Using Vacuum Assisted Resin Transfer Moulding. Open J. Compos. Mater..

[8] Andrew J.J., Dhakal H.N. (2022). Sustainable Biobased Composites for Advanced Applications: Recent Trends and Future Opportunities—A Critical Review. Compos. Part C Open Access.

[9] Benyettou R., Amroune S., Slamani M., Seki Y., Dufresne A., Jawaid M., Alamery S. (2022). Assessment of Induced Delamination Drilling of Natural Fiber Reinforced Composites: A Statistical Analysis. J. Mater. Res. Technol..

[10] Belaadi A., Hamdi L., Bourchak M. (2020). Mechanical and Drilling Performance of Short Jute Fibre-Reinforced Polymer Biocomposites: Statistical Approach. Int. J. Adv. Manuf. Technol..

[11] Varma M., Chandran S., Vijay Kumar V., Suyambulingam I., Siengchin S. (2024). A Comprehensive Review on the Machining and Joining Characteristics of Natural Fiber-Reinforced Polymeric Composites. Polym. Compos..

[12] Kroisová D., Dvořáčková Š., Knap A., Knápek T. (2023). Destruction of Carbon and Glass Fibers during Chip Machining of Composite Systems. Polymers.

[13] Raj S., Dhas J., Jesuthanam C. (2020). Challenges on Machining Characteristics of Natural Fiber-Reinforced Composites—A Review. J. Reinf. Plast. Compos..

[14] Belaadi A., Boumaaza M., Amroune S., Bourchak M. (2020). Mechanical Characterisation and Optimization of Delamination Factor in Drilling Bidirectional Jute Fibre-Reinforced Polymer Biocomposites. Int. J. Adv. Manuf. Technol..

[15] Tabet Z., Belaadi A., Boumaaza M., Bourchak M. (2021). Drilling of a Bidirectional Jute Fibre and Cork-Reinforced Polymer Biosandwich Structure: ANN and RSM Approaches for Modelling and Optimization. Int. J. Adv. Manuf. Technol..

[16] Boopathi S., Balasubramani V., Kumar R.S. (2023). Influences of Various Natural Fibers on the Mechanical and Drilling Characteristics of Coir-Fiber-Based Hybrid Epoxy Composites. Eng. Res. Express.

[17] Elhadi A., Amroune S., Slamani M., Arslane M., Jawaid M. (2024). Assessment and Analysis of Drilling-Induced Damage in Jute/Palm Date Fiber-Reinforced Polyester Hybrid Composite. Biomass Convers. Biorefinery.

[18] Malik K., Ahmad F., Gunister E. (2021). Drilling Performance of Natural Fiber Reinforced Polymer Composites: A Review. J. Nat. Fibers.

[19] Lu M.M., Fuentes C.A., Van Vuure A.W. (2022). Moisture Sorption and Swelling of Flax Fibre and Flax Fibre Composites. Compos. Part B Eng..

[20] Vacková T., Kroisová D., Spatenka P. (2009). Water Desorption Kinetics of Polymer Composites with Cellulose Fibers as Filler. J. Macromol. Sci..

